# Improving drug response prediction by integrating multiple data sources: matrix factorization, kernel and network-based approaches

**DOI:** 10.1093/bib/bbz153

**Published:** 2019-12-14

**Authors:** Betül Güvenç Paltun, Hiroshi Mamitsuka, Samuel Kaski

**Affiliations:** 1 Department of Computer Science, Helsinki Institute for Information Technology HIIT, Aalto University, Helsinki, Finland; 2 Bioinformatics Center, Institute for Chemical Research, Kyoto University, Kyoto, Japan

**Keywords:** personalized medicine, machine learning, drug response prediction, bioinformatics, heterogeneous data integration

## Abstract

Predicting the response of cancer cell lines to specific drugs is one of the central problems in personalized medicine, where the cell lines show diverse characteristics. Researchers have developed a variety of computational methods to discover associations between drugs and cell lines, and improved drug sensitivity analyses by integrating heterogeneous biological data. However, choosing informative data sources and methods that can incorporate multiple sources efficiently is the challenging part of successful analysis in personalized medicine. The reason is that finding decisive factors of cancer and developing methods that can overcome the problems of integrating data, such as differences in data structures and data complexities, are difficult. In this review, we summarize recent advances in data integration-based machine learning for drug response prediction, by categorizing methods as matrix factorization-based, kernel-based and network-based methods. We also present a short description of relevant databases used as a benchmark in drug response prediction analyses, followed by providing a brief discussion of challenges faced in integrating and interpreting data from multiple sources. Finally, we address the advantages of combining multiple heterogeneous data sources on drug sensitivity analysis by showing an experimental comparison. **Contact:**  betul.guvenc@aalto.fi

**Keypoints**

$\bullet $ Integrative analysis of drug response prediction is an essential part of personalized medicine; however, choosing informative data sources and the method that can incorporate multi-view sources is challenging.
$\bullet $ We review recent machine learning approaches solving integrative drug response prediction problem in three categories: matrix factorization-based, kernel-based and network-based methods.
$\bullet $ Understanding multi-view side data characteristics and effects on drug responses is one of the critical criteria of successful integrative drug response prediction.
$\bullet $ The predicted performance can be improved by integrating more informative data types.
$\bullet $ We conclude that the predictive approach should be selected consistent with different types of domain-specific models, data and biomedical outcomes.

## 1 Introduction

On-going technological improvements in high-throughput biology are generating increasing amounts of biological data. Thus, given the wealth of data, it is natural to take advantage of data-driven decision-making solutions in personalized medicine. One of the main computational problems of personalized medicine is to provide an understanding of cancer cell lines at the molecular level and recommend individualized therapies to patients that allow high efficacy in different cancer types by measuring drug responses [1]. As the amount of data increases, the precise computational prediction of the drug sensitivity of cancer cell lines based on molecular interactions, genomic features and chemical structures becomes essential [2, 3]. The fundamental reason is that cancer is a complex disease caused by a number of genetic mutations and somatic alterations. Machine learning (ML) algorithms are increasingly being applied to the personalized predictions of drug responses since they enable the integration of data from different sources in a statistically meaningful way to identify predictive biomarkers [4, 5]. The critical step here is how data from multiple sources are integrated to improve the prediction performance of drug responses.

In this review, we summarize recent advances that have been proposed to obtain relevant information from genomic and chemical sources for improving drug sensitivity analyses. We also compare the recent drug response prediction methods for a more profound understanding of associations between drugs and cell lines by taking advantage of side information. There are already some reviews that cover drug response prediction and data integration methods emphasizing different perspectives,

or with a special focus on a particular biological problem, to the best of our knowledge [6–11]. However, these reviews have not simultaneously (i) taken into consideration how to integrate data from different sources in drug response prediction, from the viewpoint of developing ML methods, and (ii) provided a summary of recent ML methods with the appropriate experimental comparison. Table 1 shows a detailed comparison of recent reviews.

**Table 1:** An overview and comparison of related reviews

Reviews	Drug response prediction	Data integration	Summary of recent ML methods	Experimental comparison
Computational models for predicting drug responses in cancer research [6]	✓	✓		
Algorithms for drug sensitivity prediction [7]	✓	✓		✓
A review on machine learning principles for multi-view biological data integration [8]		✓		
More is better: recent progress in multi-omics data integration methods [9]		✓	✓	
Comparison and evaluation of integrative methods for the analysis of multilevel omics data: a study based on simulated and experimental cancer data [10]		✓	✓	✓
Machine learning and feature selection for drug response prediction in precision oncology applications [11]	✓	✓	✓	
Improving drug response prediction by integrating multiple heterogeneous data sources from machine learning view point (this review)	✓	✓	✓	✓

The outline of this review is as follows: we will divide methods into three categories and summarize them as matrix factorization (MF), kernel-based and network-based methods. The reason is to give intuition that MF models are capable of learning interactions among features from different side-data sources (also called views), kernel methods capture non-linearity and fuse the similarity in higher-dimensional spaces and network-based methods are good at understanding direct and indirect associations in a heterogeneous network. Then, we provide information on relevant data sources that are commonly used to improve prediction of drug response, followed by a brief discussion of significant challenges especially when we face integrating data and evaluating predictive performance. Finally, to address the need for comparative studies, we will show an experimental comparison of drug response prediction methods underneath the data integration.

## 2 Drug sensitivity analysis

Drug sensitivity analysis is the problem of predicting the correct treatment for the right patient, for it; computational methods need to be developed to facilitate matching of patients to drugs. This problem is one of the most critical problems in the era of personalized medicine. The essential step in this task is the identification of biomarkers and developing ML algorithms for accurate drug response prediction [12]. There are some traditional ML methods focusing on this problem such as elastic net, support vector machines and random forest algorithms [3, 13–15]. However, verification of predictive biomarkers would require substantial efforts and is often expensive. The primary reason is that the cancer cells show distinct characteristics because they are influenced by diverse information from, for example, genetic, molecular and environmental sources, which makes it hard to find decisive factors. Thus, a variety of studies have been conducted with large-scale drug screenings on cell line profiles to identify predictive biomarkers [16–18].

There has been a trending demand to incorporate prior knowledge of biological systems into drug response prediction methods for improving the performance over the past decade [14]. The common understanding is that prior information provides opportunities to understand the mechanism of cancer therapy regarding the tumor progression [9, 19]. Based on this idea, various approaches have been developed for drug response prediction by integrating prior knowledge based on genomic and molecular profiles [20–22]. We summarize some of the recent improvements in this review in Section 4.

## 3 Data integration

Data integration approaches combine data from different sources in a statistically meaningful way and provide a unified view of them. It has become popular in personalized medicine recently since the need for new treatment combinations and opportunities has emerged [23, 24]. Another reason is that diseases are characterized by incredible heterogeneity, and data from only one source are not enough to capture all complexity and information to understand a disease. With the increasing data, integration methodologies demonstrated that they could achieve a more informative analysis of drug sensitivity than using a single data source by compensating missing and unreliable information in the data [25, 26].

One of the most critical challenges for data integration in personalized medicine is dealing with heterogeneous data. Data from different sources are difficult to compare because of the structure, and the majority of the current data integration systems have difficulties to overcome challenges such as different sizes, complexity and noisiness [27]. The main reason is that many of these systems are dependent on methods that have been designed to analyze one type of data, and they fail when applied to multiple data types. However, many ML algorithms have an ability to integrate diverse biological networks and can be extended to incorporate other heterogeneous data types. This review outlines the progress of computational ML models for predicting drug responses in the field of multiple heterogeneous data integration.

## 4 Drug response prediction by ML methods

**Fig. 1:**
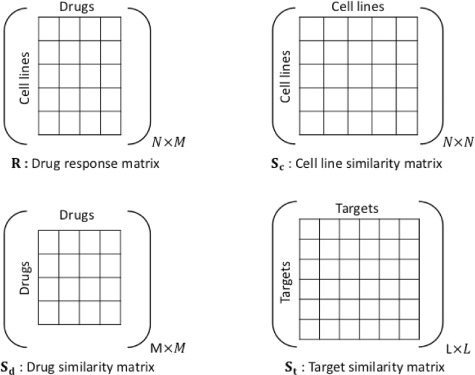
$\mathbf{R}$ is the drug response matrix, the main input. $\mathbf{S_d}$ is drug similarity matrix where the similarity between drugs *i* and *j* is denoted as $\mathbf{S_{d}}_{(i,j)}$. $\mathbf{S_{c}}$ and $\mathbf{S_{t}}$ are similarity matrices of cell lines and targets, respectively.

We briefly introduce the recent drug response prediction methods along with the integration of multiple heterogeneous data, which are categorized into three types: MF, kernel-based and network-based methods. Summary of these models and their data types are given in Tables 3 and 4. Before moving into the explanation of the methods, we define the problem that many methods have addressed. The main input is a drug response matrix $\mathbf{R} \in \mathbb{R}^{N\times M}$, in which rows correspond to cell lines and columns to drugs. Then $\mathbf{R}_{ij}$ represents the relation between entities; patient *i* and drug *j*. We consider two scenarios as an output $\widehat{\mathbf{R}}_{ij}$: (i) we can predict either missing entries in $\mathbf{R}$ and (ii) classify the cancer cell lines whether they are sensitive or resistance to given drug. Figure 1 shows the main input, possible side information and their dimensions. We use the same notations throughout this article for consistency between all methods. Table 2 shows the list of notations used in this review.

**Table 2:** The list of symbols and notations used in this paper

Symbol	Description
$C$	$C = \{c_{1},c_{2},...,c_{N}\}$; set of $N$ cell lines
$D$	$D = \{d_{1},d_{2},...,d_{M}\}$; set of $M$ drugs
$\mathbf{R}$	Drug response matrix (main input), $\mathbf{R} \in \mathbb{R}^{N \times M}$
$\widehat{R}_{ij}$	Predicted drug response value (output), (It might be real value or binary value, depends on the problem.)
$\mathbf{S}$	General notation for similarity matrix
$\mathbf{S_c}$	Cell line similarity matrix, $\mathbf{S_c} \in \mathbb{R}^{N \times N}$
$\mathbf{S_d}$	Drug similarity matrix, $\mathbf{S_d} \in \mathbb{R}^{M \times M}$
$\mathbf{S_t}$	Target similarity matrix, $\mathbf{S_t} \in \mathbb{R}^{L \times L}$
$\mathbf{U}$	Low-rank representation of cell lines, $\mathbf{U} \in \mathbb{R}^{N \times K}$
$\mathbf{V}$	Low-rank representation of drugs, $\mathbf{V} \in \mathbb{R}^{M \times K}$
$\mathbf{F}$	Feature matrix, $\mathbf{F} \in \mathbb{R}^{N \times G}$, (side information representing features separately from similarity matrices)
$\mathbf{H}$	Low-rank representation of feature matrix, $\mathbf{H} \in \mathbb{R}^{G \times K}$
$\mathbf{A}$	Projection matrix for dimensionality reduction
$\mathbf{B}$	Bias term matrix for drugs and cell lines
$\mathbf{K}$	General notation for a kernel matrix
$\mathbf{K_c}$	Cell line kernel matrix, $\mathbf{K_c}\in \mathbb{R}^{N \times N}$
$\mathbf{K_d}$	Drug kernel matrix, $\mathbf{K_d}\in \mathbb{R}^{M \times M}$
$e$	Kernel weights; $e=\{e_{1}, e_{2}, \dots , e_{P}\}$, $P$ number of matrices.
$\mathbf{T}$	Drug target interaction matrix, $\mathbf{T} \in \mathbb{R}^{M \times L}$

### 4.1 MF methods

MF has become popular mainly because of its usefulness in clustering and missing value prediction. Moreover, this approach has found its way into the domain of personalized medicine, and it is promising for modern drug discovery analyses especially because it has potential to incorporate any number of heterogeneous data [21, 28]. MF methods are considered efficient since they allow us to incorporate additional information to solve linear problems, and to extract sparse and easily interpretable factors automatically. Furthermore, they provide an additive basis to represent the data. Nevertheless, the main drawback is that they cannot capture non-linear relationships.

MF discovers hidden features underlying the interactions between entities by linear combinations of latent features. It is an unsupervised learning algorithm that decomposes a matrix into two low-rank matrices. Through this model, drug response matrix $\mathbf{R}$ can be mapped to a low-dimensional latent factor space and regarded as the product of cell lines and drugs as presented in Equation (1),

1 \begin{equation*} \mathbf{R} \approx \mathbf{U}\mathbf{V}^{\mathsf{T}}, \quad \textrm{where} \quad \mathbf{U} \in \mathbb{R}^{N \times K}, \mathbf{V} \in \mathbb{R}^{M \times K}. \end{equation*}

Matrices $\mathbf{U}$ and $\mathbf{V}$ represent the latent features of cell lines and drugs, respectively. In particular, $\widehat{\mathbf{R}}_{ij}$ results from the linear combinations of underlying latent features of cell line $i$ and drug $j$.

#### 4.1.1 SRMF

SRMF [21] was proposed as a method for drug response prediction by simultaneously incorporating drug and cell line similarity information. The main reason behind the idea is that similar drugs and similar cell lines indicate interchangeable behavior on drug responses.

**Table 3:** Summary of drug response prediction methods

Method	Prediction problem	Model	Parameter selection / Inference	Model evaluation	Performance evaluation	Note
SRMF [21]	Drug response prediction, drug repositioning	MF	Alternating minimization	10-fold CV	PCC, RMSE	Drug similarity information does not contribute to prediction performance.
HMF [26]	Drug response prediction, prediction of cancer driver genes	BMF	Gibbs sampling	10-fold CV	MSE	Automatic relevance determination eliminates the need to perform model selection.
CaDRReS [28]	Drug response prediction, drug and cell line clustering, drug-pathway associations	MF	Gradient descent	5-fold CV	SCC, NDCG	NDCG metric is preferred for ranking drugs, which is claimed useful from a clinical perspective.
QSAR [29]	Drug response prediction	KBMF	Variational inference	Nested 8-fold CV	MSE, R2, PCC	All information yields more powerful performance than drug descriptors or targets alone.
cwKBMF [25]	Drug response prediction, drug-pathway associations	KBMF	Variational inference	Nested 5-fold CV	SCC	The model recognizes component specific relationships between multiple sources and drug responses.
pairwiseMKL [22]	Drug response prediction, drug-target prediction	KRR	Conjugate gradient	Nested 10-fold CV	RMSE, PCC, F1 score	The model known as the first method for time and memory-efficient learning with multiple pairwise kernels.
Dual-Layer [30]	Drug response prediction	NBR	Sum of squared errors	LOOCV	PCC, RMSE, NRMSE	Drug similarities are more informative than cell line similarities. However, using both gives better results overall.
Stanfield’s method [20]	Drug response prediction	NBC	Random walk	LOOCV	AUC	Known as the first drug response prediction method directly integrates response data, mutation data and PPIs into a network.
HNMDRP [31]	Drug response prediction	NBC	Information flow-based algorithm	LOOCV	AUC	PPI and gene–gene correlation information have more vital role than other sources.

*Note:* MF = Matrix factorization; BMF = Bayesian matrix factorization; KBMF = Kernel Bayesian matrix factorization; KRR = Kernel ridge regression; NBR = Network based regression; NBC = Network based classification; CV = Cross validation; LOOCV = Leave-one-out cross validation; PCC = Pearson correlation coefficient; RMSE = Root mean square error; MSE = Mean square error; SCC = Spearman correlation coefficient; NDCG = Normalized discounted cumulative gain; R2 = Coefficient of determination; NRMSE = Normalized root mean squared error; AUC = Area under curve; PPI = Protein–protein interaction.

**Table 4:** Summary of data types used in drug response prediction methods

Method	Target data	Auxiliary data	Data importance	Code availability
SRMF [21]	Drug response (GDSC, CCLE)	Chemical structures (PubChem); Gene expression profile (GDSC, CCLE)	✓	https://github.com/linwang1982/SRMF
HMF [26]	Drug response (GDSC, CTRP)	Drug responses (GDSC IC50, CTRP EC50, CCLE IC50 and EC50)	✓	https://github.com/ThomasBrouwer/HMF
CaDRReS [28]	Drug response (GDSC, CCLE)	Gene expression profile (GDSC, CCLE)		https://github.com/CSB5/CaDRReS
QSAR [29]	Drug response (GDSC)	Drug–target interaction, Gene expression profile, Copy number variation, Cancer gene mutations (GDSC); Chemical structures (PubChem)	✓	https://research.cs.aalto.fi/pml/software/kbmf/
cwKBMF [25]	Drug response (GDSC, CTRP)	Gene expression profile (GDSC, CCLE) (Genomic features are divided into several views based on the prior knowledge about the pathways)	✓	https://github.com/Ammad-ud-din/cwkbmf
pairwiseMKL [22]	Drug response (GDSC)	Copy number variation, Somatic mutations, DNA methylation levels (GDSC); gene expression profiles (used in cwKBMF); 10 different molecular fingerprints	✓	https://github.com/aalto-ics-kepaco/pairwiseMKL
Dual-Layer [30]	Drug response (CCLE, CGP (GDSC))	Chemical structures (PubChem); Gene expression profile CCLE)	✓	NA
Stanfield’s method [20]	Drug response (GDSC, CCLE)	Cell line mutations (COSMIC); Protein–protein interactions (BioGRID)		http://compbio.case.edu/omics/software/drlp/index.html
HNMDRP [31]	Drug response (GDSC)	Chemical structures (PubChem); Gene expression (GDSC); Protein–protein interactions (STRING); Drug-target interaction (GDSC, KEGG, STRING)		https://github.com/USTC-HIlab/HNMDRP

*Note:* GDSC, Genomics of Drug Sensitivity in Cancer [32]; CTRP, Cancer Therapeutics Response Portal [33]; CCLE, Cancer Cell Line Encyclopedia [34]; COSMIC, The Catalogue of Somatic Mutations in Cancer (COSMIC)[35]; BioGRID [36]; STRING [37]; KEGG, Kyoto encyclopedia of genes and genomes [38]; PubChem [39].

SRMF approximates the drug response matrix by two latent factors and utilizes a weight matrix for missing values. It treats chemical structural similarity of drugs and similarity of cell lines obtained from gene expression profiles as regularization terms to avoid overfitting to training data, and imposes them to the MF model. Additionally, prior knowledge on drug and cell line similarities is used to improve prediction accuracy by minimizing the differences between the similarity of two drugs and cell lines in the latent space. The final drug response prediction model is formulated as follows:

2 \begin{equation*} \begin{aligned}[b] & \underset{\mathbf{U},\mathbf{V}}{\min} \quad || \mathbf{W} \circ (\mathbf{R}-\mathbf{U}\mathbf{V}^{\mathsf{T}})||_{F}^{2} + \lambda_{l}(||\mathbf{U}||_{F}^{2} + ||\mathbf{V}||_{F}^{2}) \\ & + \lambda_{d}(||\mathbf{S_{d}} - \mathbf{V}\mathbf{V}^{\mathsf{T}}||_{F}^{2} + \lambda_{c}(||\mathbf{S_{c}} - \mathbf{U}\mathbf{U}^{\mathsf{T}}||_{F}^{2}) \end{aligned} \end{equation*}

where $\circ $ denotes element (entry) wise product and $\mathbf{W}$ is a weight matrix that indicates whether there is a known response value. The $\lambda $’s are the regularization parameters. However, $\lambda _{d}$ and $\lambda _{c}$ can also be interpreted as weight parameters for drug and cell line similarity matrices. The model uses the alternating minimization algorithm to search for the local minimum instead of the global minimum due to the objective function not being convex.

From a graph learning perspective, when we regard the similarity as a weighted undirected graph, the most general regularization term would be graph smoothness. That is, the term $\mathbf{U}\mathbf{U}^{\mathsf{T}}$ in Equation (2) could be replaced by $\mathbf{U^{\mathsf{T}} L U}$, where $\mathbf{L}$ is the graph Laplacian, which can be generated by $\mathbf{D - S_d}$, and $\mathbf{D}$ is a diagonal matrix with its $(i,i)$ element being the sum over all elements of the $i$-th row (or columns) of $\mathbf{S_d}$. A clear drawback of Equation (2) is that the regularization part has a quadratic term that is computationally intractable, while the above graph regularizer keeps the quadratic order, which is much easier computationally.

#### 4.1.2 Hybrid matrix factorization

Bayesian hybrid matrix factorization (HMF) model [26] is a general data integration paradigm that is capable of integrating many data sets. The article answers the question of how multiple data sets can be integrated efficiently to improve predictions in the era of having many different data sets and relating entity types. It is known as the first hybrid model between MF and tri-factorization.

HMF can factorize each data into two or three latent matrices jointly, and the user can identify whether to use non-negative, semi-nonnegative or real-valued MF. The main advantage of using the probabilistic approach is the ability of handling missing values efficiently. Another benefit is that there is no need for separate model selection since Bayesian automatic relevance determination is used for seeking the exact rank in contrast to traditional MF methods. HMF builds a model that considers three types of data: (i) primary data $\mathbf{R}$, (ii) feature data $\mathbf{F}$ and (iii) similarity data $\mathbf{S}$, and each can be decomposed in different ways.

One of the common challenges in heterogeneous data integration is finding a solution that fits all data sets. This is the main reason why HMF prefers to use importance values for each data type. The likelihood is formulated by using the importance value $\alpha $, which is the power of the probabilities in Equation (3) below:

3 \begin{equation*} \begin{aligned}[b] & p(\theta | \mathbf{R, F, S}) \propto p(\theta) \times \prod_{x=1}^{X} p(\mathbf{R}^{x}| \mathbf{U}^{t_{x}}, \mathbf{V}^{x}, \mathbf{U}^{u_{x}}, \tau^{x}) ^{\alpha^{x}} \\ & \times \prod_{y=1}^{Y} p(\mathbf{F}^{y}| \mathbf{U}^{t_{y}}, \mathbf{H}^{y}, \tau^{y}) ^{\alpha^{y}} \times \prod_{z=1}^{Z} p(\mathbf{S}^{z}| \mathbf{U}^{t_{z}}, \mathbf{V}^{z}, \tau^{z}) ^{\alpha^{z}}, \end{aligned} \end{equation*}

where $\theta $ is the set of model parameters and $X, Y, Z$ are the total numbers of data sets of each type. HMF not only considers similarity matrices as side information but also features matrices, unlike SRMF, and so it is applicable to a wide range of tasks. The main distinction between HMF and multiple MF approaches [40, 41] might be the ability of using several entity types in one model.

#### 4.1.3 CaDRReS

CaDRReS [28] is a comprehensive model that attempts to solve many problems in precision medicine such as identifying drug response mechanisms, subtypes of cell-lines and drug-pathway associations by using the interaction information between drugs and cell lines. The model learns the projections of drugs and cell lines in a latent space based on a recommendation system to predict drug responses for unseen cell lines. The idea behind the preference of using a collaborative filtering technique is building a model that prioritizes information from similar drugs; hence all drugs will not have equal importance in response prediction.

The model utilizes MF to learn a ‘pharmacogenomic space’ of drugs and cell lines. The dot product of drug vector $\mathbf{v}_{j}$ and cell line vector $\mathbf{u}_{i}$ represents the interaction between the drug and the cell line. The predicted sensitivity score $ \widehat{\mathbf{R}}_{ij}$ is computed as follows:

4 \begin{equation*} \widehat{\mathbf{R}}_{ij} = \mu + b_{j}^{V} + b_{i}^{U} + \mathbf{v}_{j}. \mathbf{u}_{i} = \mu + b_{i}^{V} + b_{u}^{U} + \mathbf{v}_{j}(\mathbf{x_{u}A})^{\mathsf{T}} \end{equation*}

where $\mu $ is the overall drug response mean, and $b_{j}^{V}$, $b_{i}^{U}$ are the bias terms for drug *j* and cell line *i*, respectively. Cell line features $\mathbf{x_{u}}$ are obtained by Pearson correlation between every pair of cell lines using gene expression information. The essential part is that a transformation matrix $\mathbf{A}$ projects cell line features $\mathbf{x_{u}}$ into a latent space. This formulation can easily be seen as a decomposition of the drug response matrix $\mathbf{R}$ into biases $\mathbf{B}$ and latent factors of cell lines and drugs:

5 \begin{equation*} \widehat{\mathbf{R}} = \mathbf{B} + \mathbf{U V}^{\mathsf{T}}. \end{equation*}

CaDRReS shows the ability of predicting unseen cell-lines in contrast to SRMF because of projecting cell line features into a latent space with transformation matrix; however, it cannot provide predictions for unseen drugs.

### 4.2 Kernel-based methods

In recent years, a variety of kernel methods have been applied for drug discovery-relevant applications and have proven their ability among the best-performing approaches [42]. Kernel methods capture nonlinear patterns in the data by mapping to very high-dimensional spaces with a reasonable computational cost [43]. Besides modeling nonlinear relationships, they also offer the advantage of flexibility to work on different data types such as strings and time series. However, it may be more challenging to understand and interpret the final model than MF methods. Nonetheless, kernels can be better interpreted with the cooperation of MF and KBMF [44] can be given as an example.

We will define kernel matrix notation as $\mathbf{K}$ for the rest of the article, and similarly denote $\mathbf{K_{d}}$ and $\mathbf{K_{c}}$ for drug and cell line kernels. For example $\mathbf{K_{d}}$ can be calculated using properties of a drug in the drug response scenario with a chosen kernel function. Furthermore, kernel weights will be defined with the notation of $e=\{e_{1}, e_{2}, \dots , e_{N}\}$ for $N$ matrices.

#### 4.2.1 Integrative and personalized quantitative structure-activity relationship analysis by KBMF

Integrative and personalized quantitative structure-activity relationship (QSAR) analysis [29] is a method developed to extend traditional integrative QSAR approaches [45] by utilizing the cooperation of genomic features of cell lines and chemical drug descriptors. The motivation is that QSAR approaches are limited by concentrating on a small number of features to mainstream structural properties able to predict activity in a single cell line or a single tissue type, and hence they are not capable of solving personalized QSAR tasks.

The model builds an integrative and personalized QSAR approaches by predicting drug responses for multiple cell lines and drug efficacy for new cancer cell lines simultaneously. Bayesian MF and multiple kernel learning (MKL) paradigms cooperate to solve the drug response prediction problem. The model consists of three main parts: (i) kernel-based nonlinear dimensionality reduction, (ii) MKL that combines the view-specific factors (also called components) and (iii) MF to generate an approximated matrix by utilizing the latent factors learned from MKL. One of the essential parts is that the method determines the ‘importance weights’ for each data set to find a solution that fits all data sets. Hence, importance weights answer the question of which data type we should use for better predictive performance or how much effect does it have on the result. The probabilistic kernel QSAR model formulates the so-called composite components for drugs as follows:

6 \begin{equation*} \mathbf{V} = \sum_{m=1}^{P_{d}}e_{m} (\mathbf{A}_{d}^{\mathsf{T}}\mathbf{K}_{d,m}) = \mathbf{A}_{d}^{\mathsf{T}} \left( \sum_{m=1}^{P_{d}}e_{m}\mathbf{K}_{d,m}\right) \end{equation*}

where $P_{d}$ is the total number of drug kernels. Here $\mathbf{G_{d}} = \mathbf{A_{d}}^{\mathsf{T}}\mathbf{K_{d}}$ corresponds to dimensionality reduction (part 1) where $\mathbf{A_{d}}$ is the projection matrix. The second part combines the kernel-specific components with the kernel weights $e_{m}$. The same formula is applied for composite components of cell lines $\mathbf{U}$. The final step is MF (part 3); the approximation of drug response matrix is calculated by the multiplication of low-dimensional composite drug and cell line components obtained by kernel learning with formulation $ \widehat{\mathbf{R}}= \mathbf{U}\mathbf{V}^{\mathsf{T}}$.

**Fig. 2:**
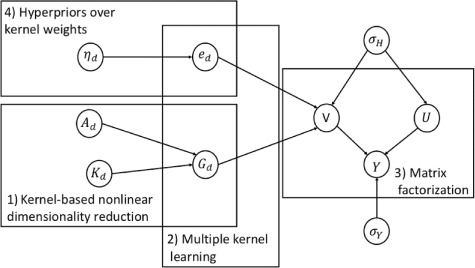
Graphical representation of cwKBMF showing four main parts of the model. The difference between personalized QSAR and cwKBMF is part 4. The same diagram can be drawn for latent factor $\mathbf{U}$.

#### 4.2.2 cwKBMF

cwKBMF [25] extended the idea of integrative and personalized QSAR by allowing selective data integration from multiple sources for predicting the response of each drug. The motivation is that integrated information does not have to be relevant to all drugs compared to in QSAR; instead, different views may be relevant for different groups of drugs. In particular, cwKBMF can learn the latent relationships between the data sources and drug responses.

Figure 2 shows a detailed graphical representation of cwKBMF with the same notations used in personalized QSAR. The model has one extra part in addition to [29], which can be seen in Figure 2 part 4 (hyperpriors over kernel weights). The main difference is in the MKL formulation, since cwKBMF requires to identify the relationship between kernels and components. Thus, the model proposes component-wise MKL, which learns the underlying factors as a combination of kernel-specific components parameterized by the component-specific kernel weights. A vital insight is that the method has the ability to understand whether kernels shared by across all components or component-specific ones. The model controls the activity of each kernel in each component by defining element-wise hyperpriors parameterized by $\eta $ over kernel weights (part 4), and this makes it possible to turn off components that are active for only a few entities. One of the disadvantages might be that the model requires normalization of drug response matrix, which can cause loss of information.

#### 4.2.3 pairwiseMKL

The pairwiseMKL [22] method was introduced as the first method for time and memory-efficient learning with multiple pairwise kernels. The primary motivation is that current MKL methods cannot feasibly scale up to the considerable number of pairwise kernels, optimize the kernel weights and train the model. By solving these challenges, pairwiseMKL builds a general approach to MKL, which can be applicable to many problems, especially pairwise learning, which involves a pair of objects, for example, drugs and their targets. The model integrates heterogeneous data sources into a single model by combining input kernels and analyzing learned kernel mixture weights for the different information sources.

There are two steps to realize the main task of constructing a pairwise kernel matrix by calculating the Kronecker product of drug and cell line kernels: (i) discovering the combination of pairwise input kernels and (ii) using these kernels to learn pairwise prediction function. First part develops an efficient Kronecker decomposition to face with the complexity comes from the centering of the pairwise kernel. New decomposition is performed by $\widehat{\mathbf{K}} = \mathbf{CKC}$, where $\mathbf{C}$ is a centering operator for $\mathbf{K}$ to generate the centerized $\widehat{\mathbf{K}}$. This decomposition helps to efficiently compute the necessary variables needed in the kernel mixture weights optimization without calculation of massive pairwise matrices. In the second stage, kernel weights obtained in the first part are used for model training by kernel ridge regression in the following form:

7 \begin{equation*} \widehat{\mathbf{R}} = \left( e_{1} \mathbf{K_{d}}^{(1)} \otimes \mathbf{K_{c}}^{(1)} +, \dots, + e_{\mathrm{P}} \mathbf{K_{d}}^{(P)} \otimes \mathbf{K_{c}}^{(P)} + \lambda \mathbf{I} \right) \pmb{\alpha} \end{equation*}

where $\lambda $ is a regularization hyper-parameter that controls the balance between training error and model complexity. $\mathbf{I}$ corresponds to identity matrix, *P* denotes the total number of sub-matrices and $\pmb{\alpha }$ is a vector of parameters obtained by the learning algorithm. Examples of kernels are kernels created by using gene expression and other molecular fingerprints. The method shows satisfactory results regarding memory usage, running time and prediction performance. Another advantage is that the method reveals which data kernels are more critical to prediction, by assigning them in the model.

### 4.3 Network-based methods

Network-based approaches are powerful for discovering interactions. The main advantages of using networks are the abilities to incorporate large amounts of data and to infer direct and indirect associations in a heterogeneous network with low computational complexity. However, with the increasing dimensionality of data sets, they might face additional challenges for ML tasks such as making feature selection difficult or ability to capture nonlinear relationships in comparison to kernel methods, but they reveal heterogeneous relations well.

**Fig. 3:**
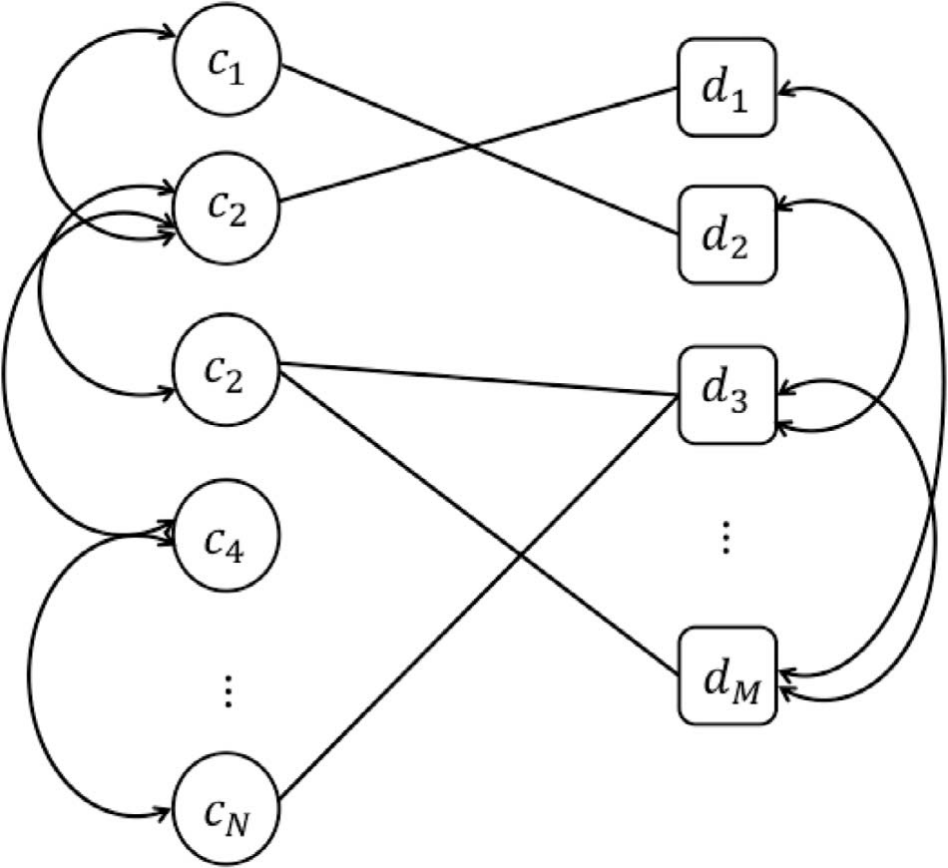
Bipartite graph representation of drug-cell line association network along with similarity networks. $d$ and $c$ represent drugs and cell lines, respectively.

Network-based approaches have already offered essential insights into disease-associated mechanisms in recent years [46, 47]. They reveal interesting relationships among subsets of cell lines and drugs. Nodes represent drugs and cell lines, edges for associations between these nodes in drug response prediction. The basic representation of drug-cell line association network can be drawn as in Figure 3. Then the score is calculated for each drug representing the probability of a cell line being sensitive (or resistant), or how much sensitive to a given drug in the network.

#### 4.3.1 Dual-layer integrated cell line-drug network model

Dual-layer integrated cell line-drug network model [30], which can use both cell line and drug similarity networks in a weighted scheme, was proposed for drug response prediction of a given cell line. The model was developed based on the observations that similar cell lines have a similar response to the same drug, and the other way around; similar chemical structures also show similar inhibitory effects over different cell lines. One of the primary outcomes from this research is that using drug similarity information is more valuable than using cell line similarity; however, using both networks gives better performance than either cell line or drug similarity network alone.

The dual-layer model has three parameters, which decide how to estimate weights on the different cell lines $w^{C}$, weigh on the various drugs ($w^{D}$) and which network will be dominated in the model $\lambda $. The advantage of having these parameters is that similar cell lines or drugs will have higher weights in their similarity networks. The model first predicts the response of a new cell line to a known drug $\widehat{S}_{C}$ and known cell line to a new drug $\widehat{S}_{D}$ separately with a linear weighted model. The linear formula predicting $\widehat{S}_{C}$ and $\widehat{S}_{D}$ can be defined as follows:

8 \begin{equation*} \widehat{S}_{C}(\mathbf{c}_{i}, \mathbf{d}_{j}) = \frac{\sum_{c_{i} \neq C}{\mathbf{R}_{ij}w^{C}(C,c_{i})}}{\sum_{c_{i} \neq C}{w^{C}(C,c_{i})}} \end{equation*}

 

9 \begin{equation*} \widehat{S}_{D}(\mathbf{c}_{i}, \mathbf{d}_{j}) = \frac{\sum_{d_{j} \neq D}{\mathbf{R}_{ij}w^{D}(D,d_{i})}}{\sum_{d_{j} \neq D}{w^{D}(D,d_{i})}} \end{equation*}

Then, $\widehat{S}_{C}$ and $\widehat{S}_{D}$ are linearly combined to make an integrated network and predict the sensitivity score of a cell line to drug, as follows:

10 \begin{equation*} \widehat{S} (\mathbf{c}_{i}, \mathbf{d}_{j}) = \lambda \widehat{S}_{D}(\mathbf{c}_{i}, \mathbf{d}_{j}) + (1-\lambda)\widehat{S}_{C}(\mathbf{c}_{i}, \mathbf{d}_{j}). \end{equation*}

The main advantage of the dual-layer model over integrative QSAR [29] is that the most similar cell lines or drugs will have similar responses and contribute much more to the prediction than the others. Another interesting comparison is that the dual-layer model shows drug similarity information has a valuable effect on prediction while SRMF results show otherwise, which might be because of parameter selection.

**Fig. 4:**
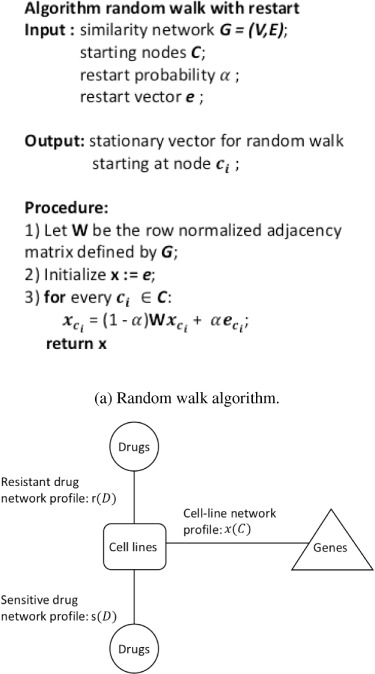
Resistance and sensitive drug network profiles contain resistant and sensitive cell lines separately for each drug. Cell line network contains the information from PPI network and cell line mutations. It is computed by performing a random walk with restart (RWR) for each cell line and drug pairs.

#### 4.3.2 Drug response prediction as a link prediction problem

Data-integrated drug response prediction problem is formulated as a link prediction in [20]. The main idea is to make feature selection easier by utilizing functional links while the number of genes is larger than samples. The model creates three network profiles, one for cell lines and two for drugs: (i) cell line network profile $\mathbf{x(c)}$, (ii) resistant network profile $\mathbf{r(d)}$ and (iii) sensitive network profile $\mathbf{s(d)}$. These profiles represent the proximity of mutated genes to each cell line and drug pair to observe how mutation information will influence the association of drugs and cell lines.

Profiles are acquired through a random walk with restarts (RWR) on a network consisting of edges representing mutations of genes, and interactions between proteins. Links representing the sensitivity or resistance of cell lines to drugs can be predicted by using associations between network profiles. Figure 4 shows RWR pseudo algorithm and the graph representation of network profiles. Sensitivity $\sigma (\mathbf{c}_{i}, \mathbf{d}_{j})$ and resistance scores $\rho (\mathbf{c}_{i}, \mathbf{d}_{j})$ of cell line-drug pair are computed by Pearson correlations between drug and cell line network profiles. The difference between these scores is utilized to assess the likelihood that a given cell line is sensitive or resistant to the given drug. Cell line-drug pair scores and final score $\delta (\mathbf{c}_{i}, \mathbf{d}_{j})$ are computed as follows:

11 \begin{equation*} \sigma(\mathbf{c}_{i}, \mathbf{d}_{j}) = corr(\mathbf{x(c}_{i}), \mathbf{s(d}_{j})) \end{equation*}

 

12 \begin{equation*} \rho(\mathbf{c}_{i}, \mathbf{d}_{j}) = corr(\mathbf{x(c}_{i}), \mathbf{r(d}_{j})) \end{equation*}

 

13 \begin{equation*} \delta(\mathbf{c}_{i}, \mathbf{d}_{j}) = \rho(\mathbf{c}_{i}, \mathbf{d}_{j}) - \sigma(\mathbf{c}_{i}, \mathbf{d}_{j}). \end{equation*}

We can interpret this formulation as a comparison of sensitive and resistant drugs through mutation information because we calculate the difference by checking if the directions of the drug vectors with cell line network are similar or not separately. Based on the final score, the cell line will have a network profile similar to the other cell lines’ network profiles that are also sensitive. This model can be used for predicting new drugs and cell lines.

#### 4.3.3 HNMDRP

HNMDRP [31] is a heterogeneous network-based approach that classifies drug responses as to whether a particular cancer cell line is sensitive or resistant. Recent network-based methods [20, 30] have already achieved promising results, and this model contributes to prediction performance by integrating protein–protein interaction (PPI) and drug-target information, which the others did not include yet.

**Fig. 5:**
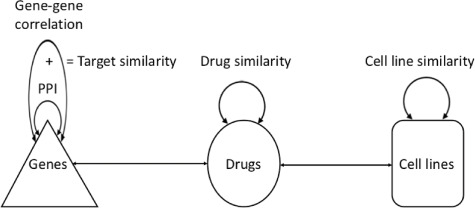
Graph representation of five different similarity and interaction network profiles. Genes, drugs and cell lines denote nodes. Target similarity network is obtained by fusing PPI information and gene–gene correlation score based on gene expression profile. For other similarity networks, the Pearson correlation coefficient is calculated between cell line profiles and drug chemical structures.

The model is constituted of five sub-networks: (i) cell line similarity, (ii) drug similarity, (iii) target similarity, (iv) drug-target interaction and (v) cell line-drug association networks. Figure 5 shows all networks, nodes and interactions. These interaction networks turn into a bipartite graph that is built according to the activity between nodes; for example, the drug response matrix is a bipartite association network between cell lines and drugs. If a cell line is sensitive to a drug the edge between them is set to 1 or otherwise to 0.

HNMDRP uses an information flow-based algorithm and a three layer network model [48] to predict drug response $\mathbf{R}^{(k+1)}$ and drug target interaction scores $\mathbf{T}^{(k+1)}$; it can be seen as an iterative algorithm:

14 \begin{equation*} \mathbf{R}^{(k+1)} = \alpha \mathbf{R}^{(k)} (\mathbf{S_{d}} \mathbf{T}^{(k)} \mathbf{S_{t}} \mathbf{T}^{(k)^{\mathsf{T}}}) + (1-\alpha)\mathbf{R}^{(0)} \end{equation*}

 

15 \begin{equation*} \mathbf{T}^{(k+1)} = \alpha (\mathbf{R}^{(k)^{\mathsf{T}}} \mathbf{S_{c}} \mathbf{R}^{(k)} \mathbf{S_{d}} ) \mathbf{T}^{(k)} + (1-\alpha)\mathbf{T}^{(0)} \end{equation*}

where $\alpha $ is the decay parameter, $\mathbf{R}^{(0)}$ denotes the initial cell line-drug associations and $\mathbf{T}^{(0)}$ the drug-target interactions. The iterative algorithm learns the final scores by utilizing similarity networks, and the current value of drug response $\mathbf{R}^{(k)}$ and drug-target information $\mathbf{T}^{(k)}$. Whenever the new scores $\mathbf{R}^{(k+1)} $ and $\mathbf{T}^{(k+1)}$ are learned, they feed into the right hand side of Equations (14) and (15).

Similarity matrices are compressed by drug response and target matrices. This is done, according to [48, 49], who modified two layer model for both predicting drug-disease and drug-target interactions. The main advantage is to be able to predict drug responses and target interactions at the same time. However, the drawback compared to [20, 30] is that the iterative algorithm cannot predict new interactions, for unseen drugs or cell lines.

## 5 Data sources

We present related data sets that are commonly used as benchmarks to evaluate drug response prediction methods.

### 5.1 Drug sensitivity data

Drug response values measure the effectiveness of a drug on a cell line at different concentrations. These measurements are summarized by metrics such as IC50 (concentration of a drug required for 50% inhibition), EC50 (concentration of a drug wto reach 50% of its maximal effect) and AUC (area under the dose-response curve) values, which are based on estimating the cell count of a treated condition, compared to an untreated control. There are three main publicly available resources for investigating drug responses, where some of the entries are missing. The statistics of these sources can be found in Table 5.

**Table 5:** Statistics of three commonly used public data resources for the development of drug sensitivity analysis methods

	GDSC	CTRP v2	CCLE
Number of experiments	>200 K	>300 K	>11 K
Number of tissue types	30	25	38
Total number of cell lines	1001	1107	1457
Number of cell lines tested on drug screening	990	887	947
Number of drugs tested on drug screening	265	544	24
Gene expression	✓		✓
Copy number variation	✓		✓
DNA methylation	✓		✓
Mutation	✓		✓

*Note:* GDSC, Genomics of Drug Sensitivity in Cancer [32]; CTRP, Cancer Therapeutics Response Portal [33]; CCLE, Cancer Cell Line Encyclopedia [34].

$\bullet $ Genomics of Drug Sensitivity in Cancer (GDSC) [32] is a collaborative project of Wellcome Trust Sanger Institute and Massachusetts General Hospital Cancer Center that combines genomic data and drug activity data. It is one of the most extensive resources on drug sensitivity in cancer cell lines, which screens more than 1000 human cell lines in a range of anti-cancer therapeutics. The current release of GDSC drug screening data contains drug responses to approximately 300 anticancer drugs across 990 cell lines.
$\bullet $ Cancer Therapeutics Response Portal (CTRP) [33] was developed by the Center for the Science of Therapeutics at the Broad Institute to screen a large panel of cancer cell lines. CTRP drug sensitivity data summarize drug responses between each cell line and drug pair using EC50 and AUC values. CTRPv2 [50] is an extended version of the CTRP project and currently known as the largest pharmacological drug screening source providing sensitivity measurements of 544 drugs on almost 900 cell lines.
$\bullet $ Cancer Cell Line Encyclopedia (CCLE) [34] is a collaborative project of the Broad Institute and the Novartis Institutes for Biomedical Research. The current version of the drug sensitivity data contains genomic data from approximately 950 human cancer cell lines against 24 anticancer drugs by allowing large-scale comparative analysis. The data include a smaller number of drugs compared to others.

The GDSC and CCLE resources not only provide drug sensitivity data but also omics data including gene expression (i.e. transcriptomic), genetic variants such as mutations and CNVs in the genome and DNA methylation data. All these data sources can be used as auxiliary information in drug response prediction models.

### 5.2 Gene expression data for drug response prediction

Gene expression is the process of determining which instructions are used to synthesize gene products. The expression level indicates the approximate amount of genetic transcription under specific circumstances or in a specific type of a cell. There is compelling evidence that gene expression information can be used to predict molecular biomarkers and drug responses to anticancer therapies [51, 52]. There are some benchmark gene expression sources that can be integrated into drug response data efficiently:

$\bullet $ CCLE [34] gene expression data were quantified by Affymetrix U133 Plus 2.0 arrays. The raw data were converted to a single value for each probe set by the robust multi-array average (RMA) approach, which is log2 transformed and then quantile-normalized.
$\bullet $ GDSC [32] gene expression data were measured by Affymetrix Human Genome U219 Array, and normalized by using RMA. Some further data processing have been applied to remove batch effects caused by growth properties.
$\bullet $ NCI-60 [53] offers a large number of omics data profiles across 60 human tumor cell lines derived from 9 different cancer tissues. This is known as the largest compound library. The expression data were obtained by integrating probes from five platforms. The probe values were first transformed to z-scores then the average score was determined for each gene for each cell line.

CCLE and GDSC projects are the commonly used sources containing gene expression levels from next-generation sequencing data for a large number of cancer cell lines. They provide more extensive coverage in terms of tissue types compared to NCI-60, thus more preferable in drug sensitivity cases.

### 5.3 Drug similarity

The chemical similarity is usually used to identify compounds sharing similar biological activity based on the structural similarity between compounds in drug discovery. The typical drug properties are as follows: (i) chemical fingerprints of drugs that capture the occurrence of fragments; (ii) 1D, 2D and 3D molecular descriptors that encode chemical composition, topology and 3D shape and functionality; and (iii) VolSurf is known as a 3D descriptor focusing on spatial properties of the drugs, which can easily be calculated by Molecular Operating Environment software [54]. The commonly used database for the chemical structural information for each drug is PubChem [39] that contains validated chemical information for 19 million unique compounds contributed from a large number of organizations. The database generates binary substructure fingerprints for chemical structures, which can be used for similarity neighboring and searching.

There are two popular computational chemical similarity tools that have been developed to link the structural properties of drugs to their biological capacity:

$\bullet $ PADEL [55] currently calculates 1875 molecular descriptors and 12 types of fingerprints mainly using The Chemistry Development Kit.
$\bullet $ The PubChem Score Matrix Service [39] can compute matrices of 2D and 3D similarity scores for a given set of compounds effortlessly for PubChem compound database identifiers.

### 5.4 Cell line similarity

Cell line similarity network derived from cell lines and tumors is the most frequently used side information in drug response prediction. The main reason is that cell lines with similar profiles tend to be within the same cancer type that has similar responses to a given drug. Thus, gene expression and copy number variation profiles have become a popular and critical information to characterize both the similarity and dissimilarity between cell lines [56]. The conventional way to calculate cell line similarities is based on three stages: (i) represent each cell line as a vector of omics features such as expression values, (ii) calculate the correlation coefficient between two represented vectors to obtain the similarity and (iii) repeat the procedure for every pair of cell lines to construct a similarity network.

## 6 Challenges

This section discusses two challenges in the computational prediction of drug responses, which earlier reviews did not mention explicitly.

1) **Data set weights**: One of the most critical challenges for data integration in drug sensitivity analysis is dealing with heterogeneous sources. Data from different sources are difficult to integrate because of the input data type, dimensionality, noise ratio and complexity. Moreover, the majority of the data-integrating drug response prediction models fail to identify the degree of relatedness between side information and target data, since they assume each side-information sources to have a binary relationship with drug response data, that is they are either relevant or not. However, if side data and the target source are dissimilar, the methods may discover a solution that fits one data set much better, and cause weak predictions for the other one [26]. For this reason, using data importance or gathering highly correlated data sets might ensure that the method will find a solution that better fits all data sets. Another common approach is identifying predictive genomic and molecular features to decrease the dominance of side information by using feature selection [57–59].

2) **Evaluation of prediction models**: An other crucial challenge in drug response prediction is the selection of performance evaluation methods. If we consider the baseline of different drugs might have a very diverse range of activity, there might be several convenient ways of evaluating prediction performance. Most drug sensitivity analyses concentrate on the correlation between actual and predicted values as a measure of efficiency [2, 34]. This approach is supported by the idea of similar gene expression or drug profiles having significantly higher drug sensitivity correlations. Another proposition claims that the correlation of drug responses between actual and predicted values might overestimate the prediction performance, so focusing on evaluation metrics for specific drugs such as drug-averaged correlation scores or drug averaged RMSE might be a better evaluation technique [21]. On the other hand, the relative order of drugs can be more crucial than the absolute values of drug responses especially for the clinical settings, because of the batch effects of different experiments [28]. These findings demonstrate that we need to consider many factors such as data, biomedical outcomes and domain-specific models when we decide the performance evaluation approach.

**Table 6:** Prediction performance of SRMF, pairwiseMKL, HNMDRP and baseline approach

	RMSE	PCC	AUC
SRMF	**1.437**	**0.899**	**0.932**
pairwiseMKL	1.690	0.856	0.908
HNMDRP	2.117	0.815	0.882
Baseline (overall mean prediction)	3.270	–	–

**Fig. 6:**
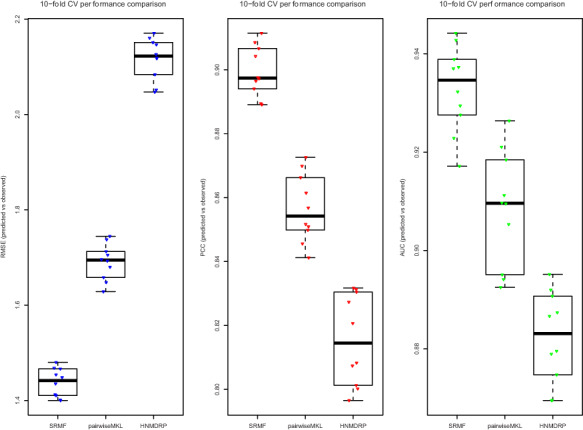
Performance comparison of predicted and observed activity of SRMF, pairwiseMKL and HNMDRP using three different evaluation measures based on 10-fold cross-validation experiments. The SRMF approach has the best cross-validated prediction performance over all evaluation methods.

## 7 Empirical comparison

We compared the performance of state-of-the-art drug response prediction methods, which were reviewed above. One model was chosen from each of three categories, which are MF-based, kernel-based and network-based methods. We considered using the latest method from each category, and comparing all methods with more general performance evaluation criteria, which are applicable to all methods, such as correlation coefficient or mean square error. However, the exception is SRMF from the first group, because, in these evaluation scores, SRMF outperformed the latest method of MF category, which is CaDRReS. We did not include other methods to comparison from similar reason, which is previously done comparisons between methods. Additionally, we wanted to compare the methods with a common baseline approach; taking mean of the training drug response data as a prediction for the unobserved drug responses. So entirely, we selected the following methods: baseline approach, SRMF from MF-based methods, pairwiseMKL from kernel-based methods and HNMDRP from network-based methods.

### 7.1 Data

We used only three types of data: drug response, cell line and drug similarity data, which are the ‘maximum’ number of data that satisfy all methods we compared. We selected the data from the sources used in pairwiseMKL whose experiments include many data sources. We only used:

$\bullet $ GDSC [32] drug response data in the form of normalized IC50 values consisting of 124 drugs and 124 human cancer cell lines,
$\bullet $ drug kernels computed by Tanimoto kernels using PubChem [39] molecular fingerprints,
$\bullet $ cell line kernels obtained from gene expression measurements by calculating Gaussian kernels.

All these data are directly suitable for SRMF and pairwiseMKL methods, but HNMDRP requires binary network data as input, as it was initially used for classification only. We applied HNMDRP here as suitable for quantitative drug response prediction to ensure their fair comparison. Furthermore, HNMDRP needs target information unlike the others since the formulation is a three-layered network. Therefore we used two uniformly distributed target data as target similarity and drug-target interaction to follow the same formulation.

### 7.2 Setting

We conducted nested 10-fold cross validation for pairwiseMKL for the hard hyper-parameters setting, following the same procedure as in original paper [22]. It requires regularization hyper-parameter $\lambda $ that controls the balance between training error and model complexity, chosen from a given set $\{ 10^{-5},10^{-4}, \dots , 10^{0} \}$. We carried out 10-fold cross-validation on SRMF and HNMDRP for the performance comparison as well. The regularization parameters $\{\lambda _{l}, \lambda _{d}, \lambda _{c}\}$ for SRMF were selected from the range provided by original paper [21] $\{2^{-3}, \dots , 2^{2}\}, \{2^{-5}, \dots , 2^{1}, 2^{0}\}, \{2^{-5}, \dots , 2^{1}, 2^{0}\}$ and the dimensionality was set to the same value as 45 for the MF. In HNMDRP, the decay factor parameter $\alpha $ was chosen in the range of $0$ to $1$ as given. The same training and test folds were used for the performance evaluation of all methods.

### 7.3 Results

Table 6 shows the results of the comparison of average predictive performances in the form of Pearson correlation coefficient (PCC), root mean square error (RMSE) and area under curve (AUC) between the actual and predicted drug sensitivity scores of the baseline method, SRMF, pairwiseMKL and HNMDRP. AUC score was computed by first ordering the actual values then converting them to ordered binary classification values according to the threshold that was chosen as mean of the actual values. The detailed performance comparison based on cross-validation experiments can be found in Figure 6.

We observe that SRMF obtained the best performance, followed by pairwiseMKL, HNMDRP and baseline. One of the reasons for SRMF’s good performance might be that it uses data importance; it achieved the best prediction performance when the drug similarity weight parameter is zero. This parameter was also the same in [21] and implies that drug similarity information does not contribute to prediction performance. Even though there is a slight difference between SRMF and pairwiseMKL, pairwiseMKL might be better where the number of data sources is significantly higher because of its time and memory efficient learning algorithm. HNMDRP showed worse performance compared to SRMF and pairwiseMKL; we believe this is likely due to not using weights over data sets, which implies every data source is considered to have the same effect on prediction performance. Another reason might be that its formulation demands a three-layer network, and for the third layer, we generated uninformative target information. Therefore, we might consider HNMDRP to be preferable for scenarios where all three types of connections are available; cancer cell line, drug and target gene nodes.

## 8 Conclusion

As more biological data become available, the traditional drug development process needs to utilize new treatment combinations and opportunities for generating novel models. Currently, there is a large number of cancer-related resources that cover disease information such as genotypes, phenotypes and their associations. It is natural to integrate these multiple data sources to create more accurate models. However, the selection of the auxiliary information to improve drug response prediction of cancer cell lines is one of the challenging parts of personalized medicine since cancer associates with many factors, including phenotypes, environmental exposures, drugs and chemical molecules, and hence it is hard to find out which one is more causative. ML methods are becoming a crucial element of modern biomedical research in this phase. There is a demand to develop methods that can integrate data from many different biomedical sources efficiently and understand what kind of effects they have on the prediction of drug responses. Thus, many methods have been developed using different types of side information for high efficiency on drug sensitivity analyses in recent years. In this review, we discussed several of the latest ML approaches that can be implemented to perform robust integrative analyses on drug response prediction problem.

We conducted an experiment comparing three methods belonging to different categories using the same data sets. In this experiment, we aimed to identify subsets of features explaining relationships between drugs and cell lines by utilizing side information. Based on the results, MF and kernel-based methods are better to detect underlying factors of drug response data by using side information from the similarity of cell lines and drugs. The network-based method is least capable of identifying the factors; however, it might show better performance to understand indirect relations such as between drug response and similarity matrices. On the other hand, choosing a method is intrinsically tied to designing the experiments. Plenty of experiments have already been done, and we should be able to use this experience to design new ones in an automated way. For example, meta-learning could fit here; it is also called ‘learning to learn’ as a way to choose a method or hyper-parameters, in other words, make specific experimental design choices [60]. Moreover, if more informative data sources can be incorporated in these models, the predictive performance might be improved. Another future direction might be predicting the response of drug combinations by integrating side information because drug combination therapy could provide an effective strategy to overcome drug resistance and incorporating prior knowledge might increase the prediction accuracy [61–63]. As the data increase, more challenges will arise, such as the redundancy between the predictive profiles, or big data problems that require carefully chosen feature selection methods. Deep learning methods have gained popularity in recent years for drug discovery and might be good direction for high dimensionality problems [64–66].

There will be a need to develop more efficient computational methods for different cancer diseases as expectations increase and opportunities emerge with the increasing data. By utilizing different types of molecular and genomic data, we can make more accurate and personalized choices for drug treatment. On-going developments demonstrate that ML methods are promising and have an exciting future for biomedical data integration, especially for the drug response prediction problem. The fundamental part here is the predictive approach, which should be selected to be consistent with different types of domain-specific models, data and biomedical outcomes, to cape with high heterogeneity between cell lines and primary patient tumors.

## Supplementary Material

suppl_data_bbz153

## Notes

**Betül Güvenç Paltun** is a PhD candidate at Aalto University. Her current research interests include the application of machine learning techniques and bioinformatics.

## Notes

**Hiroshi Mamitsuka** is a joint professor at the Bioinformatics Center, Institute for Chemical Research, Kyoto University and Aalto University. His research interests include machine learning, data mining and their applications in bioinformatics and chemoinformatics.

## Notes

**Samuel Kaski** is a professor at Aalto University. His research interests are machine learning and its applications in AI, computational biology, digital health, user interaction and brain signal analysis.
