# Supplementary material for: Improving drug response prediction by integrating multiple data sources: matrix factorization, kernel and network-based approaches
Source: Brief Bioinform. 2019 Dec 14;22(1):346–59. doi: 10.1093/bib/bbz153 (PMC7820853; doi:10.1093/bib/bbz153)
Supplement: suppl_data_bbz153 [file suppl_data_bbz153.zip › biographical_notev1.docx]

**AUTHORS BIOGRAPHY**

Betül Güvenç Paltun is a Ph.D. candidate at Aalto University. Her current research interests include the application of machine learning techniques and bioinformatics.

Hiroshi Mamitsuka is a professor at the Bioinformatics Center, Institute for Chemical Research, Kyoto University. His research interests include machine learning, data mining and their applications in bioinformatics and chemoinformatics.

Samuel Kaski is a professor at Aalto University. His research interests are machine learning and its applications in AI, computational biology, digital health, user interaction, and brain signal analysis.
