# Supplementary material for: Improving drug response prediction by integrating multiple data sources: matrix factorization, kernel and network-based approaches
Source: Brief Bioinform. 2019 Dec 14;22(1):346–59. doi: 10.1093/bib/bbz153 (PMC7820853; doi:10.1093/bib/bbz153)
Supplement: suppl_data_bbz153 [file suppl_data_bbz153.zip › keypoints.docx]

**KEY POINTS**

- Integrative analysis of drug response prediction is an essential part of personalized medicine, however choosing informative data sources and the method that can incorporate multi-view sources are challenging.
- We review recent machine learning approaches solving integrative drug response prediction problem in three categories: matrix factorization-based, kernel-based and network-based methods.
- Understanding multi-view side data characteristics and effects on drug responses is one of the critical criteria of successful integrative drug response prediction.
- The predicted performance can be improved by integrating more informative data types.
- We conclude that the predictive approach should be selected consistent with different types of domain-specific models, data, and biomedical outcomes.
